# Editorial: Neuropsychiatric and neurodegenerative aspects of acute and long COVID

**DOI:** 10.3389/fnmol.2023.1343930

**Published:** 2023-12-12

**Authors:** Abbas F. Almulla, Hussein K. Al-Hakeim

**Affiliations:** ^1^Department of Psychiatry, Faculty of Medicine, Chulalongkorn University, Bangkok, Thailand; ^2^King Chulalongkorn Memorial Hospital, The Thai Red Cross Society, Bangkok, Thailand; ^3^Medical Laboratory Technology Department, College of Medical Technology, The Islamic University, Najaf, Iraq; ^4^Department of Chemistry, Faculty of Science, University of Kufa, Kufa, Iraq

**Keywords:** COVID-19, neuropsychiatric disorders, neurodegenerative diseases, neuroinflammation, long COVID, chronic fatigue syndrome, biomarkers, neuro-immune

The escalating prevalence of central nervous system (CNS) symptoms during acute stage and 3–6 months after coronavirus disease (COVID-19), termed “Long COVID disease,” is a growing global health issue. These symptoms include chronic fatigue, mood disorders, cognitive deficits, sleep problems, and various physical symptoms like autonomic dysfunction and muscle pain. A comprehensive symptom profile (named physio-affective phenome), developed using machine learning (Maes, 2022), encapsulates these manifestations in acute (Al-Jassas et al., 2022) and Long COVID cases (Al-Hadrawi et al., 2022), highlighting the complex impact of the virus on the CNS.

The etiopathophysiology of CNS comorbidities in COVID-19, particularly in Long COVID, remains unclear. Recent studies, however, have identified key factors contributing to these conditions. Notably, evidence points to increased neuroimmune-inflammation, oxidative and nitrosative stress (Al-Hakeim et al., 2022), persistent SARS-CoV-2 infection (Vojdani et al., 2023), and autoimmune reactions (Almulla et al., 2023b). These findings have spurred the construction of the current Research Topic encompassing articles, reviews, and case reports, aimed at deepening our understanding of the biological mechanisms driving psychiatric and neurodegenerative comorbidities in acute and Long COVID.

Individuals with acute and Long COVID disease face an increased risk of developing neurodegenerative diseases like Alzheimer's Disease (AD) (Li et al., 2022). A significant contribution to this topic is an article by Trampuž et al., which investigates the common microRNA (miRNA) patterns between COVID-19 and neurodegenerative diseases, aiming to uncover shared biological pathways. The research identified 98 miRNAs common to COVID-19 and major neurodegenerative diseases, with hsa-miR-34a and hsa-miR-132 highlighted as key biomarkers due to their dysregulation in both conditions. Additionally, hsa-miR-155, upregulated in COVID-19, is also implicated in neurodegenerative processes such as AD, amyotrophic lateral sclerosis (ALS), Huntington's disease (HD), multiple sclerosis (MS), and Parkinson's disease (PD). The study also found 746 genes with significant interactions with these miRNAs.

Target enrichment analysis highlighted the significant role of KEGG and Reactome pathways in processes like signaling, cancer, transcription, and infection, with neuroinflammation as a common factor. This research indicates that miRNAs common to COVID-19 and neurodegenerative diseases could predict neurodegeneration in COVID-19 patients and might be targeted for drug development. Understanding these miRNA profiles is crucial for identifying COVID-19 patients at increased risk of neurodegenerative disorders and developing preventive or mitigative therapies. However, the clinical application of these findings necessitates further validation.

A case report by Hu et al. has been presented to the current Research Topic, which delves into the potential role of SARS-CoV-2 infection in initiating or exacerbating demyelinating diseases in the CNS. This is illustrated through a case study of a 31-year-old female patient diagnosed with relapsing-remitting multiple sclerosis (RRMS), who developed tumefactive demyelinating lesions (TDLs) following a COVID-19 infection. After the onset of TDLs post-infection, the patient received immunotherapy, specifically glucocorticoid pulses, which significantly improved clinical and radiological aspects. Subsequently, she began treatment with teriflunomide, a disease-modifying therapy (DMT).

Two months after starting teriflunomide, the patient's condition continued to deteriorate, as shown by imaging diagnostic tools. This case underscores the progression from MS to TDLs post-SARS-CoV-2 infection and highlights the limited efficacy of DMT in this context. It illustrates the complexities in diagnosing and treating such cases and suggests that SARS-CoV-2 may exacerbate demyelinating diseases in the CNS. The patient's RRMS evolved into TDLs following the infection, with teriflunomide failing to halt disease progression significantly. This transition may be linked to pre-existing MS, heightened immune reactivity, and susceptibility, indicating that SARS-CoV-2 could trigger a relapse or transformation in MS.

In an animal-based study by Xu et al., the potential neurotoxic effects of a non-neutralizing antibody, anti-S1-111 IgG, which targets the SARS-CoV-2 spike protein, were examined. The findings revealed that immunization with anti-S1-111 IgG led to increased levels of this antibody in the serum and brain homogenates of the mice. This increase was associated with heightened activation of microglia and astrocytes in the hippocampus, leading to behavioral changes indicative of psychomotor disturbances, such as impaired sensorimotor gating and reduced spontaneous activity. These changes are probably the consequences of activated neuroimmune-inflammatory pathways which confirm the role of the former pathways in inducing CNS-related symptoms in acute (Al-Jassas et al., 2022) and Long COVID disease (Almulla et al., 2023a).

Further transcriptome analysis linked these behavioral alterations to the upregulation of genes related to synaptic plasticity and mental disorders. The study suggests that the anti-S1-111 IgG, induced by the SARS-CoV-2 spike protein, can affect the CNS, particularly when the blood-brain barrier (BBB) is weakened, potentially causing neurological symptoms. These findings highlight the importance of preventing the production of such antibodies to mitigate COVID-19-related neuropsychiatric issues, especially in vulnerable individuals with compromised BBB.

The fourth study on this topic by Al-Hakeim et al. focused on examining the association between the physio-affective phenome (see above), in Long COVID, and the activity of the Tryptophan Catabolite (TRYCAT) pathway. The study's findings demonstrated that a set of biomarkers linked to Long COVID, including C-reactive protein (CRP), the kynurenine/tryptophan (KYN/TRY) ratio, and insulin resistance, along with acute COVID-19 indicators like peripheral blood oxygen saturation (SpO_2_) and peak body temperature (PBT), explained a significant portion (around 40%−41%) of the variance in the physio-affective phenome of Long COVID.

The results underscore the pivotal role of inflammatory processes during acute COVID-19 and subsequent immune-inflammatory responses in determining the physio-affective outcomes in Long COVID patients. The study indicates that the physio-affective phenome of Long COVID results from the inflammatory responses occurring during the disease's acute and extended phases. Additionally, it highlights the notable influence of decreased plasma TRY levels and increased KYN on these effects. It is worth mentioning that a recent meta-analysis showed lowered TRY levels, upregulated TRYCAT pathway and increased KYN levels in patients with acute COVID (Almulla et al., 2022). These results suggest an ongoing immune system stimulation due to the acute stage of illness leading to persistent TRYCAT pathway activation.

In summary, the present topic underscores the significant neuropsychiatric and neurodegenerative impacts of COVID-19. It stresses the need for an integrated approach in managing the long-term influences of the virus, particularly its potential to exacerbate or induce neurological disorders. The discovery of shared microRNA patterns between COVID-19 and neurodegenerative diseases offers promising avenues for early detection and treatment. This body of work deepens our understanding of COVID-19's CNS implications and emphasizes the importance of continued research and clinical innovation to address these enduring challenges.

## Author contributions

AA: Conceptualization, Funding acquisition, Investigation, Methodology, Project administration, Supervision, Validation, Writing—original draft, Writing—review & editing. HA-H: Investigation, Resources, Writing—review & editing.
